# Residual range‐based quenching correction for Gafchromic EBT3 film in proton therapy patient‐specific QA

**DOI:** 10.1002/acm2.70376

**Published:** 2025-11-18

**Authors:** Ting‐Chun Lin, Hsiu‐Ting Hsu, Yu‐Fen Chen, Yu‐Rou Chiou, Ji‐An Liang, An‐Cheng Shiau

**Affiliations:** ^1^ Graduate Institute of Biomedical Sciences China Medical University Taichung Taiwan; ^2^ Department of Radiation Oncology China Medical University Hospital Taichung Taiwan; ^3^ Department of Medicine China Medical University Taichung Taiwan; ^4^ Department of Biomedical Imaging and Radiological Science China Medical University Taichung Taiwan

**Keywords:** film dosimetry, gafchromic EBT3, LET quenching, patient‐specific QA, proton therapy, residual range

## Abstract

**Background:**

Gafchromic EBT3 film is a widely used dosimeter in radiotherapy due to its high spatial resolution and near tissue equivalence. However, its application in proton therapy is limited by the linear energy transfer (LET) quenching effect, particularly in the Bragg peak region.

**Purpose:**

This study proposes a correction method based on residual range (*R_res_*) to quantify and mitigate this quenching effect for improved accuracy in patient‐specific quality assurance (PSQA) and related research.

**Methods:**

We defined a quenching factor (QF) as the ratio of dose measured with a Markus ionization chamber to that measured with EBT3 film. The EBT film calibration curve is established by measurements in the plateau region of the depth‐dose distribution of a monoenergetic proton reference beam. A series of proton treatment plans with varying spread‐out Bragg peaks (SOBPs) were designed to examine QF across different residual ranges. A correction function correlating *R_res_* to QF was derived. Clinical verifications of patient‐specific QA were performed by comparing EBT3 measurements, with and without QF correction, to MatriXX‐PT and Eclipse TPS calculations.

**Results:**

The EBT3 film underestimated dose by approximately 7% at an *R_res_* of 1.4 cm. A clear dependence of film response on *R_res_* was observed: as *R_res_* decreased, the degree of under‐response increased. A correction function was established based on the relationship between *R_res_* and QF, enabling accurate dose reconstruction. The dose map measured using QF‐calibrated EBT3 film showed good agreement with the TPS‐calculated data, and the discrepancies between the EBT3 film measurements, MatriXX‐PT measurements, and TPS calculations were significantly reduced in regions with smaller *R_res_* values.

**Conclusion:**

We proposed and validated a clinically applicable correction function for EBT3 film quenching in proton PSQA. This approach offers a practical and cost‐effective solution for both clinical and research applications involving proton dosimetry.

## 1 INTRODUCTION

The delivery technique in spot‐scanned proton therapy, with presence of sharp dose gradients resulting from intensity modulation, can improve target conformity and healthy tissue sparing.^1^, ^2^, ^3^ Measurement of such planned dose distributions with a high degree of spatial resolution is necessary. The Gafchromic EBT3 film (Ashland, Bridgewater, New Jersey, USA), which offers high spatial resolution and near‐tissue equivalence, has become increasingly used for two‐dimensional dose distribution measurements in radiation dosimetry.^4^, ^5^, ^6^, ^7^, ^8^, ^9^, ^10^, ^11^ Compared to commercial ion chamber arrays, EBT3 films offer significant advantages for both clinical and experimental applications, including in vivo and in vitro dose measurements,^12^, ^13^, ^14^ assessments in steep dose gradient regions, and dosimetry involving heterogeneous or prosthetic materials.^15^

In clinical practice, EBT3 films are particularly valuable for patient‐specific quality assurance (PSQA), while in research settings, they serve as useful tools for evaluating complex scenarios such as dose perturbations caused by or other surgical modifications.^16^, ^17^, ^18^, ^19^ However, one limitation that has impeded their broader application in proton therapy is the quenching effect: a reduction in film response under high linear energy transfer (LET) conditions, particularly in the Bragg peak region.

The quenching effect, first noted by Martišíková et al., results from non‐uniform energy deposition within the film's active layer.^20^ As protons decelerate near the end of their range, LET increases, leading to a lower polymerization response in the film and consequently an underestimation of dose. Studies have reported under‐responses of up to 20% in the Bragg peak region for protons, and even larger discrepancies for heavier ions such as carbon.^21^, ^22^, ^23^, ^24^ To correct for this, existing methods in the literature typically involve estimating the average LET at the measurement position and applying a LET‐dependent correction factor.^25^, ^26^, ^27^, ^28^ While effective, such methods are computationally intensive and may be impractical in routine clinical settings, particularly for PSQA, where simplicity and speed are essential.

We therefore aimed to develop a correction method that is both effective and easy to implement in clinical practice. Our approach leverages the relationship between quenching and residual range (*R_res_*), defined as the distance between the depth of measurement and *R_p_* (the practical range), which is the depth at which the absorbed dose beyond the Bragg peak or SOBP falls to 10% of its maximum value.^29^ In the IAEA TRS‐398 protocol, *R_res_* is used as the beam quality index for reference dosimetry of proton beams.^29^ Since beam quality is related to LET, *R_res_* can serve as an indicator of the beam quality (i.e., the average LET) at a certain depth in the therapeutic beam. Since *R_res_* and LET are closely related, and *R_res_* is easily measurable, we hypothesized that quenching could be corrected via an empirical function of *R_res_*.

In this study, we designed a series of proton treatment plans and performed measurements at different residual ranges to systematically quantify the under‐response of EBT3 film and derive a quenching correction function based on *R_res_*. We validated the method through clinical comparisons and demonstrated its utility in routine proton PSQA and potential research applications. Gamma analysis^30^ was used for PSQA planar dose comparisons.

## 2 MATERIALS AND METHODS

### 2.1 Quenching factor and film calibration

The quenching factor (QF) was defined as the ratio of dose measured with an ionization chamber to that measured with EBT3 film:

$$QF=\frac{\mathrm{Dos}e_{Markus}}{\mathrm{Dos}e_{EBT3}}$$

The EBT3 film calibration curve (netOD vs. dose) was established using measurements in the plateau region, 2 cm depth of the depth‐dose distribution of a 160 MeV, 10 × 10 cm^2^ monoenergetic field. This depth was consistent with the determination of absorbed dose to water according to the IAEA TRS‐398 protocol. An advanced Markus chamber (PTW, Freiburg, Germany) was used for absorbed dose measurements.

Since the EBT3 film response in the plateau region of depth‐dose curve is independent of the initial incident proton energy,^22^ and when proton energy exceeds 140 MeV the variation in absolute dose at the calibration point is less than 0.8%, a 160 MeV proton beam was selected to establish the calibration curve to reduce ionization chamber measurement uncertainty. The EBT3 films were cut into 4 × 4 cm^2^ pieces and placed at the isocenter, 2 cm depth in Plastic Water (CIRS, Norfolk, Virginia, USA), perpendicular to the incident beam for each irradiation. Calibration doses of 48.0, 96.1, 192.3, and 286.9 cGy were used, adjusted according to the PSQA plan dose.

### 2.2 Plan design and irradiation setup

Irradiations were performed using a spot‐scanning beam delivery on a Varian ProBeam 360° proton system. The treatment planning system (TPS) was Varian Eclipse v16.1.

For QF evaluation, proton treatment plans were designed with a 10×10 cm^2^ field size and spread‐out Bragg peak (SOBP) widths of 3, 5, 10, and 15 cm. The treatment depth (middle of the SOBP) was set at 10 cm at the isocenter, with the beam delivered perpendicularly. Based on the SOBP width, film and chamber measurements were performed at positions within the SOBP for residual ranges of 1.6, 2.6, 3.4, 5.4, 8.4, 9.4, 11.4, and 13.4 cm.

To validate the QF correction method, five patients who had previously undergone routine PSQA measurements using the MatriXX‐PT system were selected. Treatment fields with lower gamma passing rates were chosen for EBT3 film PSQA measurements. The cancer sites included the head and neck, brain, prostate, liver, and pelvis. Gamma criteria were set to 3 mm/3%. The processing of the EBT3 film calibration curve was identical to that used in the QF evaluation.

For each PSQA field, based on the TPS dose distribution, the maximum *R_res_* and the *R_res_* at the center of the field dose distribution were determined by measuring from the dose measurement plane to the position of dose *R_p_*. These values were used for QF correction in EBT3 film PSQA measurements. The acquired EBT3 film images were analyzed with ImageJ (NIH, Bethesda, Meryland, USA) for QF evaluation, while PSQA analysis was performed using FilmQA Pro (Ashland, Bridgewater, New Jersey, USA).^31^

### 2.3 Film processing

For PSQA measurements, the entire 8″ × 10″ EBT3 film was used. For QF evaluation measurements and calibration curve establishment, films were cut into 4 × 4 cm^2^ pieces for single‐point uniform dose measurements. Films were scanned 24 h post‐exposure with an Epson Expression 12000XL flatbed scanner (Epson, Suwa, Japan) in transmission mode and at a resolution of 72 dpi. To minimize spatial non‐uniformity in scanner response and lateral response artifacts (LRAs), a template was used to position the film at the center of the scanner bed. Triple channel 48‐bit RGB mode (16 bits per channel) was used without color correction, and scans were saved in TIFF format. Raw pixel values from the color channel were converted into a net optical density value (netOD):

$$\text{netOD}={\text{OD}}_{\mathrm{irradiated}}-{\text{OD}}_{\mathrm{unirradiated}}$$

For each measurement, set of films for the dose calibration curve was also prepared. After each experimental irradiation, irradiations for establishing the dose calibration curve were performed to ensure that the measurement films of that batch shared identical background conditions.

We initially analyzed the EBT3 films using both red and green channels in 48‐bit RGB mode. Comparison with ionization chamber data showed that the green channel provided more consistent and stable response. Therefore, subsequent dose conversion analyses were conducted using the green channel.

## 3 RESULTS

### 3.1 Calibration curve of EBT3 film to net optic density

**FIGURE 1:**
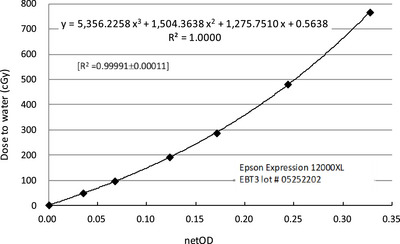
Dose to water as a function of the net optical density change for proton beam of 160 MeV with corresponding calibration fitting function.

The calibration curve for the EBT3 film (lot #05252202) exhibited a strong relationship between the net optical density (OD) and the measured dose. A third‐order polynomial model was used for curve fitting, demonstrating an excellent correlation, with a coefficient of determination of *R* ^2^ = 0.99991 ± 0.00011 (Figure 1). This result confirms the high accuracy and consistency of the film response within the evaluated dose range.

### 3.2 Depth‐dose discrepancy demonstrating the quenching effect in EBT3 film

**FIGURE 2:**
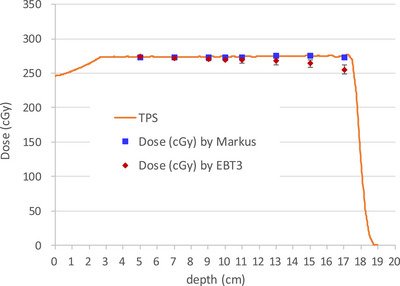
Depth‐dose curve of a 10 × 10 cm^2^ proton field with a 15 cm SOBP, measured at different depths using EBT3 film (red diamonds, mean ± STD) and an advanced Markus chamber (blue squares), overlaid with the corresponding TPS dose profile (orange line).

As shown in Figure 2, the dose measurements obtained using the ionization chamber were consistent with TPS calculations. However, the EBT3 film measurements showed a tendency to underestimate dose as *R_res_* decreased, with a notably insufficient response near the distal end of the SOBP. This finding is consistent with the known quenching effect in high‐LET regions, where the film's sensitivity decreases.

### 3.3 Development of a quenching correction function based on residual range

**FIGURE 3:**
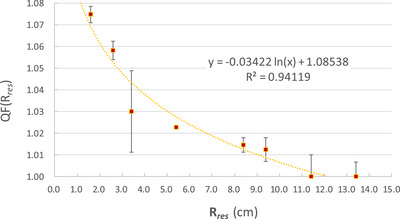
Quenching factor (QF, mean ± STD) of EBT3 film plotted as a function of residual range (*R_res_*, in cm) with corresponding fitting function. A logarithmic fitting function was applied to the data, described by the equation *y* = ‐0.03422 ln(*R_res_*) + 1.08538, with a coefficient of determination *R* ^2^ = 0.94119.

To correct the dose underestimation observed in EBT3 film measurements (Figure 2), we developed a fitting function based on the *R_res_*. As illustrated in Figure 3, the QF increased (indicating a stronger quenching effect) at smaller residual ranges, corresponding to higher LET regions near the distal edge of the SOBP. At a residual range of 1.4 cm, the QF reached approximately 1.07, indicating a 7% underestimation in the EBT3‐measured dose relative to the ion chamber. This result quantitatively confirms the LET‐related quenching effect in EBT3 film. The calculated fitting function can be applied to calibrate EBT3 measurements for more accurate proton dosimetry.

### 3.4 Gamma analysis in clinical PSQA following quenching correction

**FIGURE 4:**
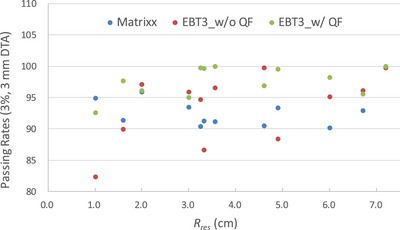
Scatter plot of PSQA gamma passing rates as a function of residual range for Matrixx/TPS, EBT3 without QF correction/TPS, and EBT3 with QF correction/TPS. EBT3_w/o: EBT3 without QF correction; EBT3_w/ QF: EBT3 with QF correction.

Table 1 shows the PSQA field measurement plane position, depth, *R_res_* value, and gamma analysis results for the five patients. Based on the dose distribution measured on the PSQA field plane, the *R_res_* values used for the QF correction were selected and highlighted with a gray background in Table 1. The EBT film measurements showed that positions with smaller *R_res_*, especially those below 2 cm, QF correction had a substantial impact on the accuracy of EBT3 dose measurements (Figure 4). Additionally, due to the 7.62 mm chambers spacing in the MatriXX‐PT, spatial dose resolution may be insufficient in steep dose gradient regions. The high spatial resolution of film dosimetry can effectively compensate for this limitation, providing more comprehensive measurement data for treatment plan dose verification.

**TABLE 1:** Comparison of PSQA gamma passing rates between Matrixx/TPS and EBT3/TPS.

				*R*_res_ (cm)		*QF* (quenching factor)		Passing rates (3%, 3 mm DTA)		
Treatment site	Tx. field no.	Plane position	Depth (cm)	max	center	max	center	Matrixx	EBT3_w/o *QF*	EBT3_w/ *QF*
Head and neck	F1 (RS 5 cm)	upper	2.6	3.0	3.0	1.044	1.044	93.5	95.9	95.0
		lower	4.6	1.0	1.0	1.079	1.079	94.9	82.3	92.6
	F3 (RS 5 cm)	upper	2.6	11.7	7.2	1.001	1.017	100.0	99.8	100.0
		lower	7.6	6.7	2.2	1.019	1.054	92.9	96.1	95.6
Brain	F2 (RS 0 cm)	upper	7.1	6.0	6.0	1.022	1.022	90.2	95.1	98.2
		lower	11.1	2.0	2.0	1.057	1.057	95.9	97.1	96.1
Prostate	F1 (RS 0 cm)	upper	12.1	11.3	4.9	1.002	1.029	93.3	88.4	99.6
		lower	20.1	3.3	—	1.041	—	91.3	89.4	99.6
Liver	F2 (RS 0 cm)	upper	12.1	4.6	4.6	1.031	1.031	90.5	99.7	96.9
		lower	15.1	1.6	1.6	1.064	1.064	91.4	89.9	97.6
Pelvis	F2 (RS 0 cm)	upper	13.6	9.5	3.2	1.008	1.042	90.4	94.7	99.8
		lower	19.6	3.6	—	1.039	—	91.1	96.5	100.0

**FIGURE 5:**
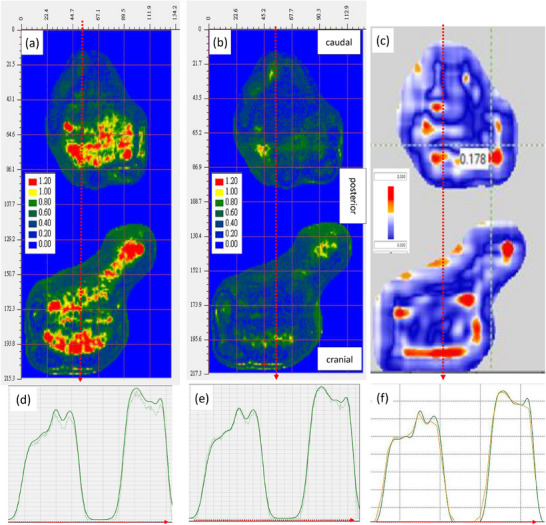
PSQA gamma analysis of the prostate case using EBT3 film and MatriXX‐PT in comparison with TPS dose distributions. (a) Gamma map of EBT3 film measurement without quenching correction. (b) Gamma map of EBT3 film after applying the QF correction derived from the residual range‐based calibration model, showing visibly reduced dose discrepancies. (c) Gamma analysis of the same case measured using MatriXX‐PT. (d), (e), and (f) Dose profiles along the positions indicated by the red dashed lines in (a), (b), and (c), respectively. In (d) and (e), the lighter lines are the EBT3 film measurements, and the darker lines are the TPS dose distributions. In (f), the orange line is the MatriXX‐PT measurement, and the dark green line is the TPS dose distribution.

Figure 5 presents gamma analysis maps of a prostate case comparing film data to TPS calculations, both with and without QF correction, alongside MatriXX‐PT results for reference. Prior to QF correction, EBT3 film data exhibited localized underestimation and poor gamma agreement with TPS. After applying the correction, the gamma passing rates improved markedly, indicating enhanced dosimetric accuracy. The corrected film results aligned more closely with both TPS and MatriXX‐PT measurements, confirming the clinical applicability of the proposed correction method. By comparing Figure 5e, f, it can be observed that MatriXX‐PT measurements may be limited by the relatively low spatial dose resolution. The measured data exhibit unsmoothed linear interpolation at the apex of the dose distribution, requiring greater caution when measuring dose distributions with high gradients. Furthermore, using the interpolation function provided by the application software that processes MatriXX‐PT measurement data results in a smoother dose distribution; however, this may cause averaging of the dose at the apex, leading to an underestimation of the actual dose at that location.

## 4 DISCUSSION

We introduced a practical, *R_res_*‐based correction method to account for LET‐related quenching in EBT3 film for clinical proton dosimetry. Unlike traditional approaches that rely on complex LET distribution calculations, our method applies *R_res_*, the distance between the measurement point and the *R_p_*, as a clinically intuitive and accessible surrogate for quenching correction. This correlation is supported by established dosimetric protocols, such as IAEA TRS‐398. The approach demonstrated high accuracy and ease of implementation, making it suitable not only for routine PSQA workflows but also for investigative research.

EBT3 films offer several different advantages over single‐point or 2D‐array ionization chambers, such as the Markus chamber and MatriXX‐PT. The Markus chamber is accurate for absolute dose measurement. However, it is too bulky for in vivo applications. The MatriXX‐PT is widely used for PSQA measurements and serves as a highly efficient, user‐friendly tool that provides reliable data in most cases. However, with the 7.62 mm spacing between its detector points, it may lack sufficient spatial dose resolution in regions with steep dose gradients. In contrast, EBT3 film is lightweight, flexible, and provides high spatial resolution, making it ideal for small fields, the presence of metal implants, or steep dose gradients. These characteristics support its use in specialized measurements, including in vivo verification or complex geometry QA.

In the early stage of this study, we referred to literature that used the red channel for film analysis under 48‐bit RGB scanning of EBT3 films. However, we observed inconsistencies between different measurement times and with ionization chamber data. Upon reviewing the literature, a study by Lloyd Smyth et al.^32^ indicated that the green channel generally exhibits lower uncertainty compared to the red channel. After switching to the green channel for analysis, the overall consistency and stability of our measurement data improved.

In general, for EBT3 film analysis at doses below 10 Gy, the red channel shows the highest dose sensitivity^22^ and is therefore commonly used for dose analysis. However, in this study, the QF correction values were within approximately 7%, and measurement stability was crucial for reliable interpretation of the experimental results. Although the green channel has slightly lower dose sensitivity than the red channel, its lower uncertainty provided more reliable data measurement.

By modeling the QF as a function of *R_res_*, our correction function significantly improved dose agreement between film and ion chamber measurements. When applied to clinical PSQA cases, QF correction consistently improved gamma passing rates in each clinical case when compared with both TPS and ion chamber‐based MatriXX‐PT measurements. This confirms the practical utility of the method in a clinical setting.

Several limitations remain. First, the correction function must be verified for each new EBT3 film batch due to inter‐lot sensitivity variations. Another limitation arises in complex treatment plans, such as head and neck cancer cases, where a single radiation field may contain a wide proton range distribution and thus varying *R_res_* values. Applying a single QF across such heterogeneous dose distributions may introduce inaccuracies. To address this, we propose a segmented correction strategy using different QF values tailored to deeper and shallower regions within the same field, which may mitigate residual discrepancies in large or irregular targets.

In summary, the *R_res_*‐based quenching correction method offers a clinically viable, efficient, and accurate solution to enhance EBT3 film dosimetry in proton therapy. With appropriate calibration and local validation, it holds promise for broader integration into QA protocols, particularly in contexts requiring high‐resolution, flexible measurement tools.

## 5 CONCLUSION

We developed and verified a residual range‐based correction function to compensate for the LET quenching effect in Gafchromic EBT3 film. This method enhances the accuracy of EBT3 film for proton PSQA and offers potential utility in various research applications, including in vivo and in vitro dose measurements, assessments in steep dose gradient regions, and dosimetry involving heterogeneous or prosthetic materials.

## AUTHOR CONTRIBUTIONS

All authors contributed to the study conception and design. Material preparation, data collection, and analysis were performed by An‐Cheng Shiau, Hsiu‐Ting Hsu, and Yu‐Fen Chen. Ting‐Chun Lin and An‐Cheng Shiau drafted the manuscript. An‐Cheng Shiau, Yu‐Rou Chiou, and Ji‐An Liang interpreted the data and commented on the manuscript. All authors read and approved the final manuscript.

## CONFLICT OF INTEREST STATEMENT

The authors declare that they have no relevant financial or non‐financial interests to disclose.

## ETHICS STATEMENT

This study was approved by the ethics committee of China Medical University Hospital (CMUH113‐REC3‐105).
